# Two cases of *TBL1XR1* heterozygous variants in children: a new splicing site variant identification and functional analysis through molecular docking and molecular dynamics simulation

**DOI:** 10.1186/s40246-025-00877-9

**Published:** 2025-12-24

**Authors:** Yaxue Xie, Ziyan Zhang, Gang Zhu, Zhichao Li, Huiling Zhang, Jiaqi Zhang, Lin Wan, Guang Yang

**Affiliations:** 1 Department of Pediatrics, The First Medical Center of Chinese PLA General Hospital, Beijing, 100853 China; 2 Senior Department of Pediatrics, The Seventh Medical Center of Chinese PLA General Hospital, Beijing, 100700 China; 3 Medical School of Chinese People’s Liberation Army, Beijing, China

**Keywords:** Pierpont syndrome, Minigene, Molecular docking, Molecular dynamics, *TBL1XR1*

## Abstract

**Background:**

Transducin β-like 1 X-linked receptor 1 (TBL1XR1) protein is an important component of NCoR/SMRT complex. The variants of *TBL1XR1* are associated with Pierpont syndrome (PS) and developmental delay (DD). This study aimed to discover new *TBL1XR1* variants, their clinical manifestations, and protein-level changes.

**Methods:**

Whole-exome sequencing was used to identify patients with *TBL1XR1* variants in 2024. Minigene assay was used to investigate specific splice site, which was further validated by Sanger sequencing. Structural changes in the TBL1XR1 protein were analyzed using PyMOL and molecular dynamics (MD) simulations. Potential binding partners were predicted via Genecards, STRING, and Cytoscape, while molecular docking was employed to assess how variants affect protein complex interactions.

**Results:**

Two novel *TBL1XR1* variants (c.1048-8_1049del and c.865-7A>G) were identified in two patients. Patient 1 exhibits global developmental delay (GDD), while patient 2 displays with facial dysmorphism and autism spectrum disorder. c.865-7A>G is a non-canonical splicing variant causing abnormal mRNA splicing. SpliceAI and RDDC predicted its splicing pattern. Minigene analysis found a 6 bp (TCTCAG) insertion in mRNA, leading to two amino acid (SQ) insertion in the TBL1XR1 protein. Therefore, P2 was diagnosed with PS. Variant changed the local hydrogen bond network and electrostatic potential. MD simulations showed variant changed the conformation of TBL1XR1 protein. Protein–protein interaction analysis selected NCOR1 for docking with TBL1XR1. Their interaction was reduced after the insertion of SQ, which may contribute to the occurrence of PS.

**Conclusion:**

This study reported two patients manifesting with GDD and PS, which were identified with two novel variants of *TBL1XR1* (c.1048-8_1049del, p.(N350X)) and (c.865-7A>G, p.K288_T289insSQ), respectively. c.865-7A>G variant might lead to PS by reducing its interaction with NCOR1.

**Supplementary Information:**

The online version contains supplementary material available at 10.1186/s40246-025-00877-9.

## Introduction

*TBL1XR1*, which encodes the protein transducin-beta-like-1 X-linked receptor 1, is located at 3q26.32 and composed of 18 exons [1]. TBLXR1 (or TBLR1) is a 514-amino acids protein, compromising the amino-terminal LiSH domain and carboxy-terminal tryptophan-aspartic acid 40 (WD40) domain. TBL1XR1 is a component of the nuclear receptor corepressor (N-CoR) and silencing mediator of retinoic acid and thyroid hormone receptor (SMRT) protein complex, which regulates transcription [2]. Besides, TBL1XR1 plays a critical role in recruiting β-catenin to the promoters of Wnt target genes for transcription activation [3].

According to the OMIM database, variants in *TBL1XR1* are associated with Pierpont syndrome (PS; OMIM: 602342) [4, 5]. PS is an autosomal dominant syndrome characterized by global developmental delay (GDD), distinctive facial features (e.g., frontal bossing, midface hypoplasia, depressed nasal bridge), hearing loss, and palmar/plantar fat pads [4]. Besides, a case with PS harboring a *TBL1XR1* missense variant (p.Gly237Asp) presented with both dysmorphic features and autism [6]. Heinen et al. reported a recurrent missense variant, p.Tyr446Cys, in six PS patients and proposed a dominant-negative mechanism for this mutation [5]. Furthermore, *TBL1XR1* variants have been implicated in various neurodevelopmental disorders (NDDs), including developmental delay (DD), intellectual disability (ID), and autism spectrum disorder (ASD) [7–11], which frequently co-occur [10]. DD is nearly universal in *TBL1XR1*-related disorders, with language function being most commonly affected [12].

Precursor messenger RNAs (Pre-mRNAs) splicing constitutes a crucial step in the generation of mature, translatable mRNAs during eukaryotic gene expression [13]. The conserved splice sites, namely the 5’ splice donor site and the 3’ splice acceptor site, function as key splicing signals. These sites can be identified by the spliceosome, thereby facilitating the splicing process of pre-mRNAs [14]. Genomic variants that occur outside the canonical splicing site (± 2) have the potential to induce abnormal mRNA splicing and are categorized as non-canonical splicing variants (NCSVs). In this study, we enrolled patients harboring *TBL1XR1* variants. To elucidate the abnormal splicing mechanism, we performed the minigene assay, widely regarded as the most straightforward and reliable experimental means to ascertain whether a variant impacts exon recognition and potentially gives rise to phenotypic alterations [15]. We then inferred the function of the mutant (MUT) protein by constructing tertiary structure models and conducting molecular dynamics (MD) simulations. Furthermore, we utilized bioinformatics analysis to screen for proteins that interact with TBL1XR1, leading to the identification of NCOR1. Finally, molecular docking was used to investigate the interaction between TBL1XR1 and NCOR1 following the occurrence of the variant.

## Material and methods

### Collection clinical information

Two patients with *TBL1XR1* variants detected by whole exome sequencing (WES) in the outpatient services of the First Medical Centre of the PLA General Hospital across the whole 2024 were studied. The clinical information and written informed consent were obtained from the patients. Genomic DNA was extracted from the peripheral blood leukocytes of Patient 1 and Patient 2 (P1 and P2). WES was then performed on these samples at Beijing Mygenostics Co., Ltd for Patient 1 and Berry Genomics Co., Ltd for P2, respectively. Subsequently, the sequencing reads of P1 and P2 were aligned to the Genome Reference Consortium Human 38 (GRCh38). All procedures were in accordance with the ethical standards of the Ethics Committee of the First Medical Center of the PLA General Hospital (Ethical approval: S2025-526-01) and the Declaration of Helsinki. Gesell Developmental Schedules (GDS) [16–18] is a classic international child development scale that was utilized to evaluate the mental development of this child. It examines five domains, including adaptability, gross motor, fine motor, language, and social personality. The results are expressed in terms of the developmental quotient (DQ). Based on the degree of neurodevelopmental disability, the DQ scores were divided into mild (55 ≤ DQ < 75), moderate (40 ≤ DQ < 55), severe (25 ≤ DQ < 40), and suspected (75 ≤ DQ < 84) neurodevelopmental disability. Whereas children with DQ ≥ 85 were normal.

### Bioinformatics analysis

**Fig. 1:**
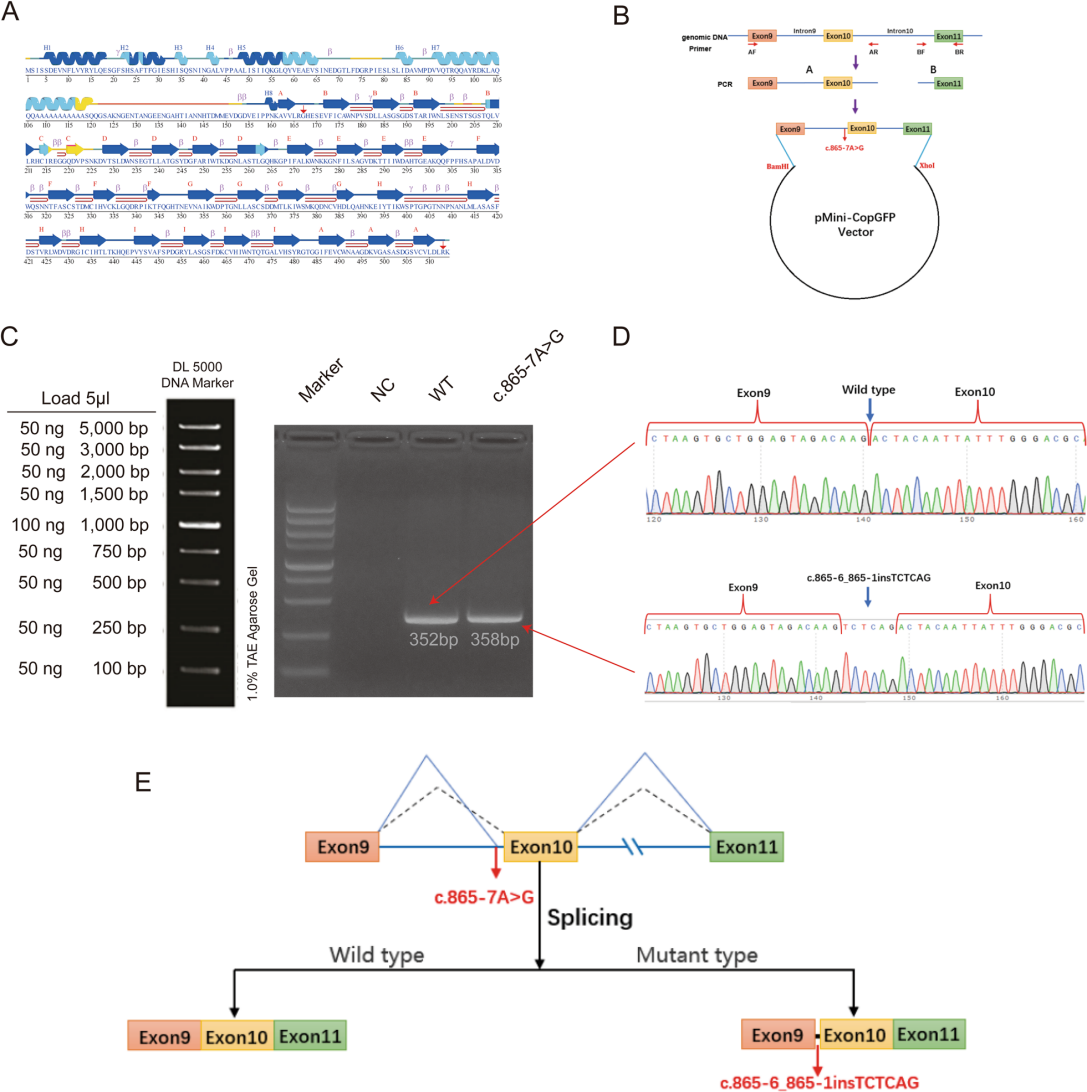
*TBL1XR1* constructs for investing the splicing pattern of c.865-7A>G in P2. **A** The secondary structure diagram was retrieved from the PDBsum server. The start and end sites of WD40 domain were labeled with red arrows. In this diagram, helical regions are marked with “H” followed by a number above the corresponding sequence, while sheets are labeled with alphabetical characters in the same position. The coloring of the diagram indicates the Alphafold confidence scores: blue and light blue signify very high and high confidence levels respectively, whereas yellow and orange denote low and very low confidence. Additionally, “β” stands for beta turn, “γ” stands for gamma turn, and a red hairpin symbolizes a beta hairpin. **B** The flow chart of plasmid construction. **C** Agarose gel electrophoresis of RT-PCR products expressed from the TBL1XR1 minigenes in HEK 293T cells. WT: 352 bp; *TBL1XR1* c.865-7A>G: 358 bp. **D** Sanger sequencing of RT-PCR for the plasmid expression. **E** Schematic of splicing pattern for WT and *TBL1XR1* c.865-7A>G. *NC* negative control, *P2* Patient 2, *RT-PCR* Reverse Transcription Polymerase Chain Reaction, *WT* wild type

Splicing patterns of the putative splicing variant was predicted using the online tools including SpliceAI (https://spliceailookup.broadinstitute.org/), and RDDC (https://rddc.tsinghua-gd.org/tool/rna-splicer). The pathogenicity of variant was predicted using the bioinformatic analysis tool-PROVEAN (http://provean.jcvi.org/index.php) [19], which could predict the functional effect of amino acid insertions and deletions (indels). The secondary structure information of TBL1XR1 was analyzed using the PDBsum server (Fig. 1A; http://www.ebi.ac.uk/pdbsum/) [20]. The wild-type (WT) TBL1XR1 structural model was retrieved from AlphaFold Protein Structure Database (v.2; https://alphafold.ebi.ac.uk/) [21] with the identifier of AF-Q9BZK7-F1. AlphaFold-predicted structures exhibit accuracy comparable to experimental structures in most cases, with root-mean-square deviation (RMSD) of predicted monomeric protein backbones approaching experimental levels [21]. The modeling of MUT TBL1XR1 was performed using the homology modeling tool SWISS-MODEL (http://www.swissmodel.expasy.org) [22], with AF-Q9BZK7-F1 as the template. This was done to ensure both WT and MUT models are derived from the same structural template, thereby minimizing interference from template differences in the analysis of mutation effects. Homology modeling is more suitable for local structural adjustments targeting specific amino acid mutations, ensuring stronger comparability with the WT structure. The most reliable 3D structure was selected based on the Global Model Quality Estimation (GMQE = 0.89) [23] and QMEANDisCo global score [24] (0.76 ± 0.05) values. Ramachandran plot [25] analysis was used to validate both the WT and MUT protein structure through the online tool PDBsum (http://www.ebi.ac.uk/thornton-srv/databases/pdbsum/Generate.html). Visualization of the structures of both the WT and MUT proteins was achieved using PyMOL software v.3.1.0 [26]. Besides, PyMol plugins provide convenient means for visualizing the hydrogen bonds (donor–acceptor distance was ≤ 3.5 Å, and the donor-hydrogen acceptor angle was ≥ 135°) and electrostatic potential [27].

### Minigene splicing assay

To validate the potential splicing effects induced by c.865-7A>G mutation, an in vitro minigene splicing assay was performed. The minigene regions of the *TBL1XR1* gene encompassing exon 9, intron 9, exon 10, intron 10, and exon 11 (Fig. 1B), were amplified from the gDNA of the WT. The forward and reverse primers were designed to include BamHI (F: 5′-AAGCTTGGTACCGAGCTCGGATCCGTAACCTTGCTAGCACCTTAGGGCAGCATA-3′; R: 5′-CATGCAAGTGGTATATCATTTAGCCTTATCCCATAACAAACAG-3′) and XhoI (F: 5′-AATGATATACCACTTGCATGTTACCCTTATCTAAAGAGG-3′; R: 5′-TTAAACGGGCCCTCTAGACTCGAGCGTATGTCCTTGGAATGTTTTAATAGGT-3′) restriction sites to promote cloning into the pMini-CopGFP vector (Fig. 1B; Beijing Hitrobio Biotechnology Co., Ltd.) using a ClonExpress II One Step Cloning Kit (Vazyme, Nanjing, China). Mutant plasmids were created by recombining the mutant fragments obtained with the mutagenesis primers TBL1XR1-MT-F (5′- TCCTTTTAGTCTCAGACTACAATTATTTGGGACGCACAT-3′) and TBL1XR1-MT-R (5′-GTCTGAGAcTAAAAGGAAAAGAAAAATAAACTCATGG-3′). Both the WT and mutant plasmids were validated by Sanger sequencing. The empty pMini-CopGFP vector served as the negative control (NC). The selected recombinant plasmids were transiently transfected into Human epithelial kidney 293 T (HEK293T) cells using Lipofectamine 2000 (Invitrogen, Carlsbad, CA, USA). Total RNA was extracted from cells that had been cultured for 48 h, subsequently reverse transcription-polymerase chain reaction (RT-PCR) was performed using MiniRT-F (5′-GGCTAACTAGAGAACCCACTGCTTA-3′) and TBL1XR1-RT-R (5′- CGTATGTCCTTGGAATGTTTTAATAG-3′) primers. The amplified Polymerase Chain Reaction (PCR) fragments were analyzed by agarose gel electrophoresis, and the isoforms were determined by Sanger sequencing.

### Molecular dynamics simulations

To explore the structural changes of the WT and MUT TBL1XR1 during molecular dynamics simulations, this study performed 3 sets of MD simulations for each protein following the steps below to avoid randomness in the trends of simulation results. The WT and MUT TBL1XR1 monomer coordinates obtained in the previous section were used as starting structures for initiating the MD simulations. GROMACS (2020.3) [28] along with the Amber99 force field were used for MD simulations. A cubic system containing TIP3P water molecules was utilized in the MD simulation [29]. Before the simulation, the system underwent 50000 steps of minimization using the steepest decent method and neutralization with counterions of NaCl. Under the NVT system (constant number of particles, volume, and temperature), the LINCS (Linear Constraint Solver) algorithm was employed to constrain the vibrations of all covalent bonds. The V-rescale thermostat was used for temperature control to maintain the system temperature at 300 K with a temperature coupling constant of 0.1 ps. The SETTLE algorithm was adopted to restrict the geometric conformation of water molecules. No positional restraints were applied to the protein atoms; only the LINCS and SETTLE algorithms were used to constrain covalent bond vibrations, allowing the protein to freely adjust its conformation during the simulation. The double-range cutoff radius for van der Waals interactions were set to 1.0 and 1.2 nm, and the long-range electrostatic interactions were treated using the Particle Mesh Ewald method [30]. During the NPT (constant number of particles, pressure, and temperature) simulation, the Parrinello-Rahman barostat was utilized for pressure control. The time constant for pressure coupling was set to 2.0 ps, and the reference pressure was set to 1 × 10^5^ Pa. All restrictions on atomic parameters and temperature settings were the same as those in the NVT simulation. After the density of the system reached the optimal state limit, a 500-ns equilibrium simulation was performed on the system. The minimum distance between the simulation box and the protein in the XYZ direction was set to 1.0 nm. A time step of 2 fs was also employed. RMSD, root-mean-square fluctuation (RMSF), radius of gyration (Rg) and solvent-accessible surface area (SASA) were calculated over the 500-ns simulation period to describe conformational changes during the simulation process.

### Protein–protein interaction

To delve deeper into the protein-protein interaction (PPI) network, we queried the Genecards database (https://www.genecards.org/) [31] using the term "Pierpont syndrome" to retrieve PS-related genes. Subsequently, protein-coding genes were all preserved to import into STRING database (http://string.db.org/) for PPI analysis and visualized using Cytoscape v.3.10.3 [32]. Proteins directly interacting with TBL1XR1 within this network were extracted and further visualized. In the visualization, the width of each edge represents the combined score of the corresponding protein.

### Molecular docking

Moreover, the interaction between WT and MUT TBL1XR1 with NCOR1 was analyzed by means of Alphafold 3.0 (AF3; https://alphafoldserver.com/) [33, 34], which could demonstrate substantially high complex interactions prediction accuracy. In addition, to validate the specificity, we include GAPDH as the negative control. The sequences of these proteins were retrieved from UniProt database in FASTA format for analysis. For the WT-NCOR1, MUT-NCOR1, and GAPDH-NCOR1 combinations, seed values of 1234567890, 987654321 and 543216789 were used, respectively. The interaction was further visualized using PyMOL software. Additionally, to gain crucial insights into the binding strength between complexes derived from AF3, Molecular Mechanics/Generalized-Born Surface Area (MM/GBSA) was computed using the HawkDock web server (http://cadd.zju.edu.cn/hawkdock/) [35]. PDBePISA (https://www.ebi.ac.uk/pdbe/pisa/) [36, 37], which was based on chemical thermodynamics, is used to explore protein interfaces, solvation free energy gain (Δi*G*) and the *p*-value of the observed solvation free energy gain (*p* < 0.5 indicates that the interface surface can be interaction-specific). PRODIGY (https://rascar.science.uu.nl/prodigy/) [38, 39], which was based on ligand-specific scoring functions, was used to predict the binding free energy (Δ*G*) and dissociation constant (*K*d).

## Results

### Patients

#### Patient 1

**Fig. 2:**
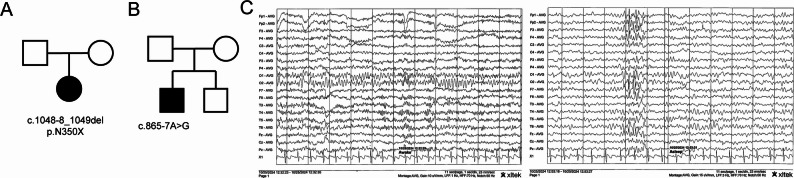
Clinical information of two patients with de novo* TBL1XR1* frameshift variants. **A** Pedigree plot of the family for P1. **B** Pedigree plot of the family for P2. **C** EEG showed the background rhythm is slowed down, and a large number of low-amplitude fast waves are interspersed in all leads; During the sleep period, a small number of low-to-medium amplitude spikes (sharp waves) and spike-slow waves are discharged in the bilateral frontopolar, frontal and anterior temporal regions. (5y4m). *EEG* electroencephalogram; P1/2: Patient 1/2

P1 is female who was born at full-term via vaginal delivery, with a birth weight of 3.4 kg (0 SD ~ 1 SD) and length of 50 cm (0 SD ~ 1 SD). Her parents, both aged 31 years, were in good health and were not bound by any consanguinity (Fig. 2A). Besides, she was diagnosed with binocular strabismus at birth and underwent surgical treatment at the age of 8 months. Her facial appearance and hearing are both normal. The child had delayed intellectual and motor development. She could sit independently at 1 year old and walk independently at 1 year and 9 months old. Currently, her walking posture is somewhat abnormal. She can run, but her coordination is poor. She can squat down and stand up, but he cannot jump. Around 1 year old, she could say " papa, mama ", but not consciously. She cannot say words and sentences. When she has a need, she will actively say reduplicated words. She can imitate speaking a little, but not clearly. She lacks social skills, has language understanding, but is poor at following complex instructions. Her response to being called sometimes is good. She cannot sing but can sway her body according to the rhythm of music. She is not stubborn. According to Table 1, the GDS score shows mild to moderate developmental delay in gross motor, and the other four domains show moderate to severe developmental delay. The brain magnetic resonance imaging (MRI) and electroencephalogram (EEG) are normal. She was diagnosed with global developmental delay. The genetic analysis outcome indicated that a novel variant within *TBL1XR1* (NC_000003.11(NM_024665.7): c.1048-8_1049del, p.N350X). This deletion caused the premature emergence of a stop codon, causing the loss of 165 amino acids and consequently impairing the normal functionality of the protein, which was classified as “pathogenic” (PVS1 + PS2 + PM2) based on American College of Medical Genetics (ACMG) guidelines [40, 41]. In all, this patient was diagnosed with GDD. This mutation was absent in the clinvar, ExAC, gnonAD and 1000G databases. She is now 4 years and 3 months old, her weight was 13 kg (-3 SD ~ -2 SD) and height was 100 cm (-2 SD ~ -1 SD). She was unable to attend school.

**Table 1:** Clinical information of two patients

Patient	P1	P2
Gender	Female	Male
Birth history	G1P1	G2P1
Age at diagnosis	11 m	6 m
Parents’ age at the time of birth	F and M: 31y	F: 28y; M: 25y
Growth parameter at birth		
Weight	3.4 kg	3 kg
Length	50 cm	49 cm
Growth parameter at present		
Weight	13 kg	18.3 kg
Length	100 cm	110 cm
Neurobehavioral phenotype		
Intellectual delay	Yes	Yes
Hypotonia	No	No
Language development delay	Yes	Yes
Seizure	No	No
Autism	No	ABC: 80 (4y7m); 79 (5y10m) CARS: 32 (4y7m)
Rett features	No	No
Schizophrenia	No	No
Brain MRI	No	No
EEG	Normal	The background rhythm is slowed down, and a large number of low-amplitude fast waves are interspersed in all leads; During the sleep period, a small number of low-to-medium amplitude spikes (sharp waves) and spike-slow waves are discharged in the bilateral frontopolar, frontal and anterior temporal regions. (5y4m)
Global developmental delay	GDS: Gross motor: 41.6, fine motor: 34.1, adaptability: 32, language: 31, social personality: 40.9 (3y7m); Gross motor: 56.9, fine motor: 41.1, adaptability: 34.8, language: 31.6, social personality: 31.6 (3y11m)	GDS: Gross motor: 26, fine motor: 39, adaptability: 35, language: 29, social personality: 32 (2y3m); Gross motor: 30.5, fine motor: 30.5, adaptability: 25.8, language: 21.1, social personality: 21.9(5y4m)
Dysmorphic features	No	Yes
Diagnosis	GDD	PS
Other	Strabismus: surgical correction	α-thalassemia (maternal)

*ABC* Autism Behavior Checklist, *CARS* Childhood Autism Rating Scale, *cm* centimetre, *F* father, *GDD* global developmental delay, *GDS* Gesell Developmental Schedules, *kg* kilogram, *M* mother, *m* months, *PS* Pierpont syndrome, *y* years old

#### Patient 2

This patient (P2) was born by vaginal delivery at 36^+4^ weeks of gestation. His parents are healthy and non-consanguineous. The father is 28 years old and the mother is 25 years old when he was born (Fig. 2B). The birth weight was 3 kg (-1 SD ~ 0 SD), the body length was 49 cm (-1 SD ~ 0 SD), and the head circumference was unknown (Table 1). He has a healthy younger brother who is now 4 years and 3 months old. He had a high forehead, narrow palpebral fissures, short hands and feet, deeply marked palmar and plantar grooves, and fat pads on the hands and feet. The proband’s motor and cognitive development was delayed. He started turning over at 1 year old, sit up at 2 years old and walked at the age of 4 years. When assessed at 5 years and 5 months old, he exhibited a lack of active language, only unconsciously said "papa, mama". He was inclined to engage in self-talk but showed no inclination or ability to interact with those around him. Eye contact was sporadic and infrequent. While he was capable of following a select few simple instructions and would respond with a smile when teased, he remained unable to indicate his need to urinate or defecate. There is no obvious stereotyped behavior. The DQs across all five domains in GDS were less than 40, which consequently signified that the child was afflicted with a severe neurodevelopmental impairment (Table 1). His Childhood Autism Rating Scale (CARS) score was 32, which falls within the range of 30 to 36, indicating that he had mild autism. Autism Behavior Checklist (ABC) at 4 years and 7 months and 5 years and 10 months indicted that there was a relatively high risk of autism for him (≥ 67). The EEG showed a slowing of the background rhythm, with a large number of low-amplitude fast waves interspersed throughout. During the sleep stage, a small number of low-to-medium amplitude sharp waves, spike-and-wave complexes were discharged in the bilateral frontal polar, frontal, and anterior temporal regions (Fig. 2C). Whereas, brain MRI was normal. In all, this patient was diagnosed with PS. His Single Nucleotide Polymorphism (SNP) results indicated a de novo heterozygous variant (*TBL1XR1*: NC_000003.12(NM_024665.7): c.865-7A>G). According to the ACMG, this variant was classified as "variant of uncertain significance" (VUS, PS2_Moderate + PP3 + PM2_Supporting), which was absent in the clinvar, ExAC, gnonAD and 1000G databases. Since this variant was located near the exon–intron junction, SpliceAI and RDDC were used to predict the potential impact on alternative splicing (Table 2 and Figure S1). As predicted by SpliceAI, the delta score of this variant for acquiring a novel acceptor was 1.00. Through RDDC, two potential abnormal splice patterns were forecasted. Splice pattern 1 involved 6 bp insertion, while splice pattern 2 lead to 61 bp deletion spanning intron 9, exon 10, and intron 10, without bringing about premature termination. His Copy Number Variation (CNV) results revealed a 15.12 Kb deletion in 16p13.3 region: 165,983-181,108 inherited from his mother. This region includes HBM, HBA2, HBA1 and HBQ1, which is associated with the pathogenesis of α-thalassemia. He is 5 years and 10 months old now, his weight was 18.3 kg (-2 SD ~ -1 SD) and height was 110 cm (-2 SD ~ -1 SD). He was unable to attend school.

**Table 2:** Genetic analysis

Patient	Position	Genotype		Prediction	
		Variant	ACMG	SpliceAI	PROVEAN
P1	chr3:177038170–177038180 (exon12)	NC_000003.12(NM_024665.7): c.1048-8_1049del, p.N350X	Pathogenic: PVS1 + PS2 + PM2	NA	NA
P2	chr3:177046196–177046196 (intron9)	NC_000003.12(NM_024665.7): c.865-7A>G	VUS: PS2_Moderate + PP3 + PM2_Supporting	Acceptor loss: 0.96; Acceptor gain: 1.00	Deleterious: −10.333

*NA* Not Available, *P1/2* patient 1/2

### Splicing study of TBL1XR1 c.865-7A>G using minigene assay

To further elucidate the abnormal splicing, we carried out a minigene analysis on both the NC, WT and the MUT. Agarose gel electrophoresis of RT-PCR products revealed no bands in the NC. The WT sample showed a single band at the size of 352 base pairs (bp), while the mutant type exhibited a single band corresponding to the size of 358 bp (Fig. 1C). Sanger sequencing divulged a normal splicing isoform in the WT and an abnormal splicing pattern in the mutant type (Fig. 1D), which was in accordance with the splicing pattern 1 (6 bp insertion, Figure S1) predicted by RDDC. The minigene analysis indicated that the c.865-7A>G substitution was capable of generating a novel acceptor splice site (GT) within intron 9 of the *TBL1XR1* gene. This led to the insertion of six bases (TCTCAG) in the TBL1XR1 cDNA possessing c.865-7A>G variant (Fig. 1E), and it was predicted to result in the insertion of Serine and Glutamine between site 288 and 289 (i.e. p.K288_T289insSQ) within the TBL1XR1 protein. To further investigate the pathogenicity of this insertion, the PROVEN tool was utilized, which indicated that the insertion was likely to be deleterious (score = -10.333) (Table 2).

### Local hydrogen bonds and electrostatic potentials changes

**Fig. 3:**
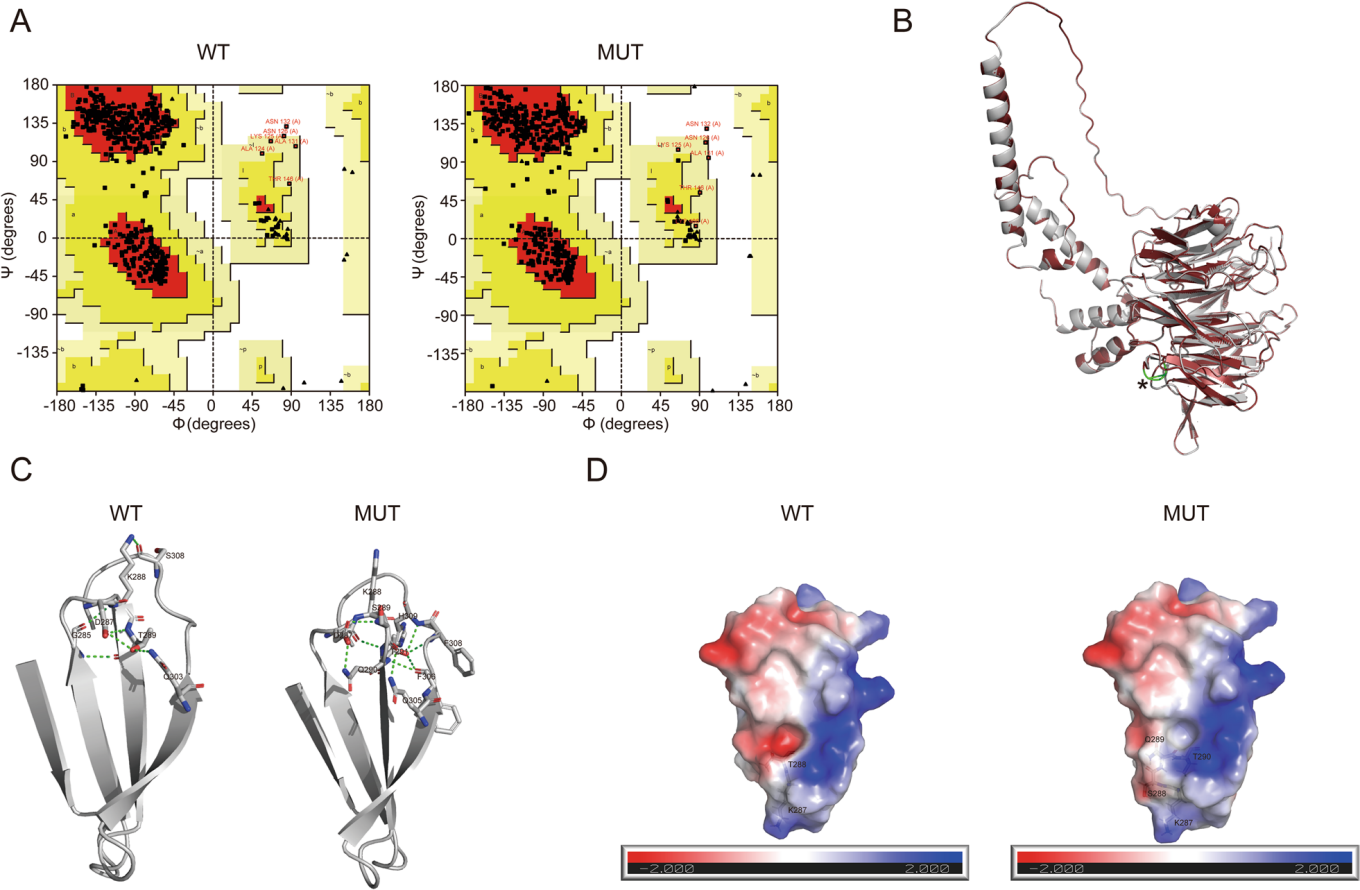
Molecular model alteration of TBL1XR1 following insertion variant. **A** Ramachandran plot for WT and MUT TBL1XR1. Number of non-glycine and non-proline residues is 453 in WT and 455 in MUT; Number of end-residues (Gly and Pro) is 2 in both WT and MUT; Number of glycine residues (shown as triangles) is 41 in both WT and MUT; Number of proline residues is 18 in both WT and MUT. Residues in most favoured regions [A, B, L] are 90.7% (411/453) in WT and 92.7% (422/455) in MUT; residues in additional allowed regions [a, b, l, p] are 7.9% (36/453) in WT and 5.9% (27/455) in MUT; residues in generously allowed regions [~ a, ~ b, ~ l, ~ p] are 1.1% (5/453) in WT and 0.9% (4/455) in MUT; residues in disallowed regions are 0.2% (1/453) in WT and 0.4% (2/455) in MUT. Amino acids with incorrect φ and ψ values are labeled in red. **B** Structure overview of TBL1XR1 p.K288_T289insSQ (dark red) superimposed with WT (gray). Two inserted amino acids were colored with green and marked with asterisk (*). **C** The network of Hbond interactions within the E-sheet of TBL1XR1. This involves K288 and T289 residues in the WT, as well as K288, S289, Q290, and T291 in the MUT. The Hbond is colored in green. **D** The local electrostatic potentials of E-sheet in WT and MUT. The electrostatic potential is display from red (−2.0 kT/e) to blue (+ 2.0 kT/e). *Hbond* hydrogen bond, *MUT* mutant, *WT* wild type

We constructed the MUT TBL1XR1 model using the SWISS-MODEL. Both the WT and MUT TBL1XR1 models were further validated via Ramachandran plots, which confirmed their good quality (residues in most favoured regions > 90%; Fig. 3A). Besides, the WT and mutant proteins were displayed in Fig. 3B. The mutation sites K288 and T289 are located in the E-sheet (Fig. 1A) and WD40 domain, which is functionally important region. Therefore, this insertion could affect the function of TBL1XR1.

We first analyzed changes in the E-sheet caused by the insertion of Serine and Glutamine, including hydrogen bonds (Hbonds) and electrostatic potentials. In WT TBL1XR1 (Fig. 3C), 2 Hbonds exist between K288 and two residues (G285 and S308), 4 Hbonds exist between T289 and three residues (G285, D287 and Q303). The insertion results in the addition of two Hbonds in the E-sheet, thereby causing a change in the protein conformation. Specifically, 2 Hbonds exist between S289 and two residues (D287 and Q305), 2 Hbonds exist between Q290 and two residues (D287 and Q305), 4 Hbonds between T291 and three residues (F306, F308 and H309). This could be attributed to that two inserted amino acids (Serine and Glutamine) are polar, introducing new hydrogen bond donors and acceptors. In the local region of E-sheets, the insertion of S and Q leads to a slight reduction in surface negative charge (Fig. 3D), possibly by reducing the exposure of negative charges through direct spatial or chemical effects.

### Molecular dynamics simulation analysis

**Fig. 4:**
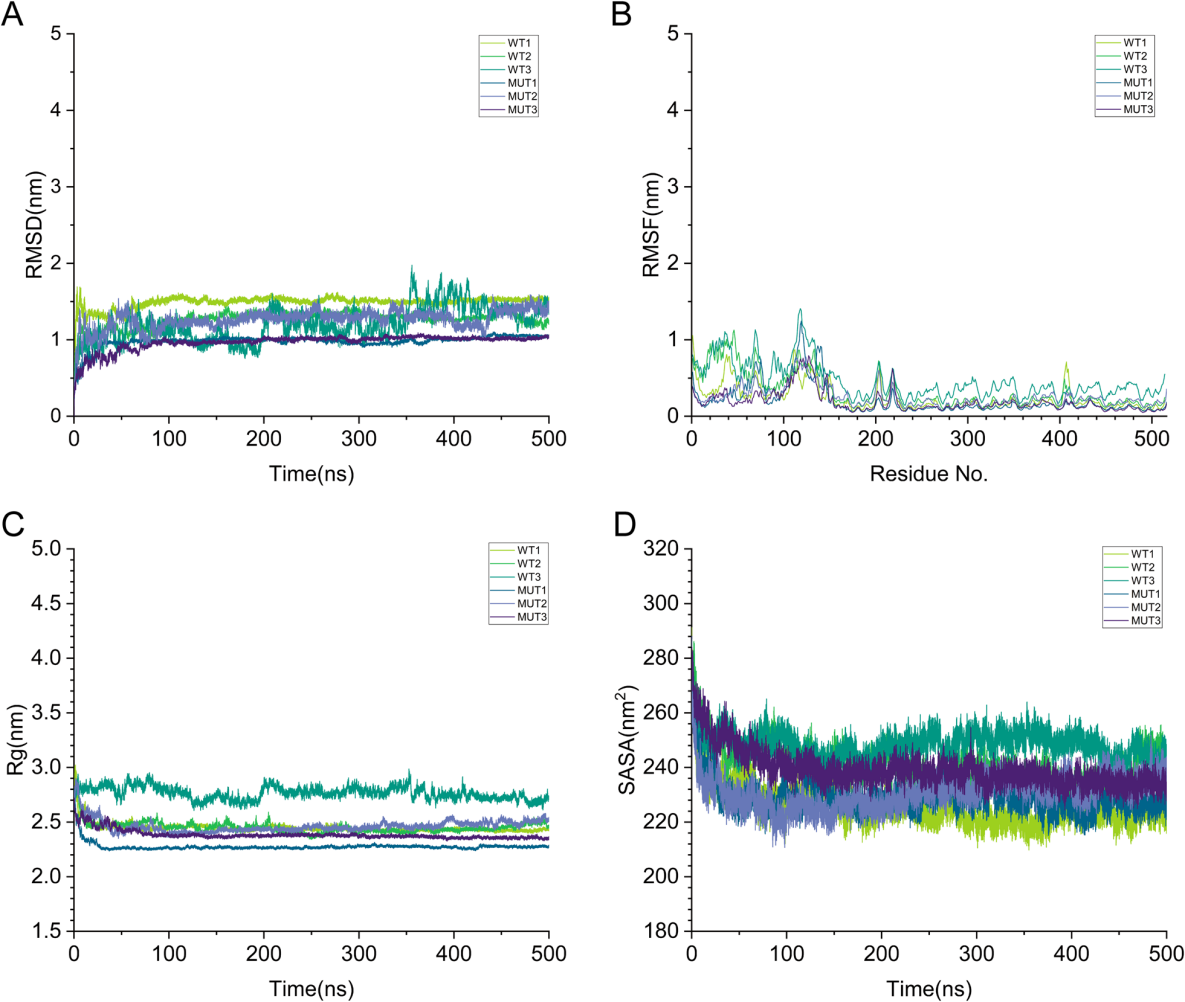
Analysis of WT and MUT TBL1XR1 molecular dynamics simulation. **A** RMSD of all atoms for the WT and MUT TBL1XR1. **B** RMSF of each residue for the WT and MUT TBL1XR1. **C** Rg of all atoms for the WT and MUT TBL1XR1. **D** SASA of each residue for the WT and MUT TBL1XR1. *RMSD* root-mean-square deviation, *RMSF* root-mean-square fluctuation, *Rg* radius of gyration, *SASA* solvent-accessible surface areas, *WT* wild-type

To further elucidate the effect of the mutation on the whole protein structure, we performed 3 sets of MD simulations (WT/MUT 1-3) for 500 ns to comprehensively explore the changes after insertion, and put them together (Fig. 4).

Throughout the simulation, the RMSD trajectory trends of WT1 and WT2 were highly consistent (Fig. 4A). They began to stabilize after ~ 150 ns, with average stable values of 1.5 ± 0.15 nm and 1.35 ± 0.2 nm, respectively, indicating that the protein maintained a relatively stable global conformation thereafter. In contrast, WT3 exhibited a more fluctuating RMSD value (1.5 ± 0.5 nm), suggesting that it might have sampled another conformational state or possessed greater conformational flexibility. Notably, no anomalies (e.g., unexpected simulation termination) were observed in WT3, confirming that its conformations still lie within the natural sampling range of the protein. MUT1 and MUT3 converged rapidly at around 30 ns and 180 ns, respectively, and stabilized at 0.9 ± 0.10 nm in the subsequent periods (Fig. 4A). However, MUT2 showed a larger fluctuation amplitude and a delayed convergence trend, with an average RMSD of 1.4 ± 0.2 nm, suggesting that it might have entered a different conformational state, reflecting the dynamic diversity of the protein.

The biological function of proteins depends on the functional domain residues and their structural fluctuation [37]. We therefore calculated RMSF to assess residue-specific fluctuations and global stability. As shown in Fig. 4B, RMSF distributions of WT1 and WT2 were highly consistent, with low flexibility in the core region and slightly higher flexibility in residues of regions such as the N-terminus, 25-35, 65-72, 115-120, 130-135, 145-150, 202-206, 218-221, and 410-415. WT3 exhibited higher flexibility in the N-terminal loop region compared to other regions. A closer observation of WT3 revealed that its RMSF change trend was basically consistent with that of WT1 and WT2, but with larger fluctuations in RMSF values, which was consistent with the greater fluctuations in RMSD, indicating a certain degree of increased flexibility and structural rearrangement. The residue fluctuation distributions of MUT1-3 were consistent (Fig. 4B). Among them, the RMSF fluctuations of MUT1 and MUT2 were basically the same in most residue regions, with the main domains remaining stable and only slight flexibility observed in partial loop regions. The flexibility fluctuations of MUT3 were slightly larger than those of MUT1 and MUT2. This altered flexibility is likely to affect TBL1XR1 conformation and its interactions with other proteins.

The Rg is a metric reflecting protein compactness [38]. As shown in Fig. 4C, the folding dynamics of the proteins throughout the MD simulations were consistent with the corresponding changes in RMSD. The differences in Rg further confirm that the insertion of SQ impacts TBL1XR1 conformation.

In order to measure the compactness of the backbone, the change in SASA was analyzed. Most WT simulations showed higher SASA values than the MUT counterparts (Fig. 4D and Table 3), which indicated the SQ insertion may disrupt interactions with other molecules by reducing overall SASA.

**Table 3:** The calculated parameters (average values ± SD) for all the systems based on 500 ns MD simulation after balance

Protein	Simulation	RMSD (nm)	Rg (nm)	SASA (nm^2^)
WT	1	1.5 ± 0.15	2.42 ± 0.05	228.00 ± 4.00
	2	1.35 ± 0.2	2.45 ± 0.10	244.00 ± 8.00
	3	1.50 ± 0.50	2.80 ± 0.10	252.00 ± 4.00
MUT	1	0.90 ± 0.10	2.30 ± 0.05	228.00 ± 8.00
	2	1.40 ± 0.20	2.40 ± 0.05	240.00 ± 8.00
	3	0.90 ± 0.10	2.53 ± 0.05	240.00 ± 8.00

*MUT* mutant, *RMSD* root-mean-square deviation, *Rg* radius of gyration, *SASA* solvent-accessible surface areas, *WT* wild-type

### Protein–protein interaction of TBL1XR1 in PS and molecular docking analysis

To further elucidate the potential mechanism underlying the pathogenesis of PS, we searched the Genecards database using the keyword “Pierpont syndrome” and obtained 135 protein-coding PS-related genes (Table S1). All genes were selected to construct the PPI network, which consisted of 111 nodes and 371 edges (Figure S2A). In this network, the proteins that have a direct connection with TBL1XR1 (n = 11) were chosen, and they were utilized to further explore the TBL1XR1-related mechanism in the pathogenesis of PS (Figure S2B and Table S2). The proteins-HDAC3, NCOR1 TBL1X and TBL1Y, which have the strongest interaction with TBL1XR1, was selected based on the combined score (0.999) (Figure S2B and Table S2).

It has been reported that HDAC3 does not interact with TBL1XR1 [42]. Additionally, TBL1/TBL1XR1 mediates tetramerization via its N-terminal LiSH domain [43]. Given that the mutation in P2 resides within the WD40 domain, and both the N- and C-termini of TBL1XR1 are known to interact with NCOR1 [42], NCOR1 was finally chosen. The mutation of TBL1XR1 in PS leads to altered interactions with other proteins rather than loss of function, indicative of a dominant-negative effect [5]. Therefore, we employed molecular docking to investigate its interactions with other components of the NCoR/SMRT complex, particularly NCOR1 in this analysis. The formed Hbonds of the complex model_0 analyzed by AF3 and binding site residues were displayed using PyMol (Figure S2 C-D and Table S3).

Moreover, the HawkDock, PDBePISA and PRODIGY web servers were employed to assess the binding affinity between WT/MUT and NCOR1. WT-NCOR1 complex exhibited a stronger binding affinity than MUT-NCOR1 complex, the detailed values were shown in Table S4. Evidently, the WT exhibits a stronger binding affinity with NCOR1, as reflected by its more negative binding free energy value. To validate the specificity of the TBL1XR1-NCOR1 interaction, we performed molecular docking between GAPDH and NCOR1, which served as a NC in this analysis (Figure S3 and Table S4).

## Discussion and conclusion

TBL1XR1 contains one LisH domain in the amino-terminal domain (4-36 aa), an F-box-like domain (41-86 aa), and eight WD40 domains in the carboxy-terminal (167–513 aa), which play an essential role in the NCoR/SMRT corepressor complex [2, 5]. NCoR/SMRT complex mediates transcriptional suppression by binding to nuclear hormone receptors. The WD40 domain of TBLR1 has a β-propeller structure, which consists of eight propeller “blades”. Each blade is composed of four anti-parallel β-sheets, and these blades are connected by β-hairpins. The blades are arranged symmetrically around the central axis, just like the staves of a barrel [44].

In our study, P1 was diagnosed with GDD. This diagnosis was established based on the absence of typical PS features, including characteristic facial dysmorphology and palmar/plantar fat pads. Additionally, P1 had normal EEG and no evidence of repetitive behaviors. Although strabismus has been reported in PS patients [45], this finding is less specific than a high forehead, underdeveloped midface, narrow palpebral fissures, anteverted nares, and palmar/plantar fat pads. It may also arise from unrelated factors, such as abnormal development of ocular muscles and can occur in various genetic disorders. Genetically, P1 harbored a *TBL1XR1* mutation resulting in the loss of 165 amino acids within the evolutionarily conserved WD40 domain. WD40 repeats are known to mediate protein-protein interactions [46], suggesting that this variant may destroy binding to interacting partners, thereby contributing to neurological dysfunction. The loss of function mutations in TBL1XR1 contribute to the pathogenesis of NDD [10]. According to ACMG criteria, this variant was classified as "Pathogenic". Collectively, these findings indicate that the TBL1XR1 variant (NC_000003.11(NM_024665.7): c.1048-8_1049del, p.N350X) is likely responsible for the development of GDD in P1.

P2 was diagnosed with PS based on clinical scale evaluations, facial dysmorphisms, delayed development, and a minigene assay of the *TBL1XR1* intronic variant NC_000003.12(NM_024665.7): c.865-7A>G, which confirmed this NCSV as pathogenic for aberrant mRNA splicing. Electrophoresis revealed a 6-bp insertion in TBL1XR1 mRNA, resulting in the insertion of two amino acids (Serine and Glutamine) between positions 288 and 289 of the TBL1XR1 protein. This mutation is located within the third WD40 domain, and was predicted to be deleterious by means of PROVEAN. It has been proposed that variants may exert deleterious effects by altering protein structural features, such as disrupting intramolecular Hbond networks and modifying electrostatic potential distributions [47]. We therefore investigated changes in local Hbond networks and electrostatic potential landscapes. Our results demonstrate that the insertion of two neutral polar amino acids induces structural modifications in the protein. Specifically, this insertion impacts intramolecular Hbond formation, leading to the addition of two Hbonds within the β-sheet, and slightly decreases the negative charge in the local electrostatic surface potential of TBL1XR1. In addition, we performed MD simulation to investigate the impact of mutation on the whole protein structure. According to Fig. 4 and Table 3, these analyses revealed that the TBL1XR1 variant induces conformational changes, alters residue flexibility, and reduces the SASA.

Amino acid residues at positions 288 and 289 are part of the WD40 domain, which mediates PPIs. Leveraging this, we mined online databases to identify potential TBL1XR1 interactors in PS pathogenesis. NCOR1, a core component of the NCoR/SMRT repressor complex, was finally selected as the candidate. TBL1XR1-driven PS pathogenesis involves a dominant-negative effect, where mutant proteins disrupt interactions with binding partners [5]. Yoon et al. showed that the first three WD40 repeats of TBL1XR1 are critical for binding to the RD4 domain of NCOR1 [42]. Thus, we employed AF3 to perform molecular docking to assess how the mutation affects TBL1XR1-NCOR1 interactions. Collectively, this variant impairs the stability of the TBL1XR1-NCOR1 complex through two key mechanisms: first, by disrupting Hbond formation with NCOR1 (Figure S2C-D); second, via subtle surface charge alterations-including a slight increase in negative charge on the adjacent "top surface" and a mild elevation in positive charge on the side surface (Figure S2E-F). These changes are accompanied by reduced absolute binding energy with NCOR1, as evidenced by the analyses of web servers (Table S4). The reduced interaction between TBL1XR1 and NCOR1 could impair transcriptional regulation, which, as supported by previous studies, may disrupt the MAPK cascade signaling pathway [48] and thyroid hormone metabolism [49, 50], thereby contributing to the pathogenesis of PS.

In conclusion, this study reports two novel *TBL1XR1* variants with distinct phenotypic manifestations. Using in silico approaches, both variants were predicted to be pathogenic, as they disrupt the structure and function of the TBL1XR1 protein. Additionally, this work expands the genotypic and phenotypic spectrum associated with TBL1XR1-related diseases. Studies have found no significant clinical or phenotypic differences between TBL1XR1 missense, truncating mutations, deletions, CNVs, or SNVs/indels [10, 51]. Additionally, analysis of 43 patients with WD40 domain mutations (encompassing PS, MRD-41, and unclassified phenotypes) revealed no clear genotype–phenotype correlation [45]. In summary, *TBL1XR1* variants are linked to a broad phenotypic spectrum, with even same-locus variants causing distinct manifestations due to differing mutated amino acids [52], necessitating further investigation into genotype–phenotype relationships.

However, the study has certain limitations. First, the TBL1XR1-NCOR1 docking model was constructed without incorporating experimental constraints (e.g., NMR data or mutation-based validation), rendering it a hypothetical structure. Due to the lack of direct experimental evidence-such as small-angle X-ray scattering (SAXS) data or mutation analyses-this model only reflects potential interaction modes rather than the authentic binding characteristics under physiological conditions. To address these limitations, we plan to systematically validate the reliability of the docking model through follow-up experiments, including co-immunoprecipitation, site-directed mutagenesis combined with surface plasmon resonance, and SAXS. These experiments will help overcome the constraints of current computational simulations. Furthermore, the precise pathogenic mechanisms underlying the phenotypes of PS and GDD remain elusive. In-depth experimental investigations are needed to elucidate how *TBL1XR1* variants impact associated signaling pathways.

## Supplementary Information

Supplementary material 1: Table S1. Pierpont syndrome-related protein coding genes from Genecards database.

Supplementary material 2: Table S2. Directly interacted proteins with TBL1XR1 based on the String database. Proteins were ranked according to the combined score. The node colored with red was finally chosen.

Supplementary material 3: Table S3. Amino acid residues involved in Hbond formation between WT/MUT TBL1XR1 and NCOR1, as well as between GAPDH and NCOR1, along with the corresponding Hbond distances. Hbond: hydrogen bond; MUT: mutant; WT: wild type.

Supplementary material 4: Table S4. Results of the binding affinity assessed by the HawkDock, PDBePISA and PRODIGY web servers. ELE: Electrostatic energy; GB: Generalized Born energy; *K*d: dissociation constant; SA: Solvent Accessible Surface Area energy; TOTAL: binding free energy; VDW: Van der Waals energy; Δi*G*: solvation free energy gain; Δ*G*: binding free energy.

Supplementary material 5: Figure S1. Two splice patterns predicted by RDDC tool. Pattern 1 with 6 bp insertion and pattern 2 with exon 10 (61 bp) skipping. bp: base pairs. Figure S2. Molecular docking between TBL1XR1 and NCOR1. A. Interactive PPI network obtained from Genecards and String databases. It includes 111 nodes and 371 edges. Each node represents PS-related proteins, each edge represents the associated interaction. Pale green indicates proteins that have indirect interaction with TBL1XR1; light yellow indicates proteins that have direct interaction with TBL1XR1; orange indicates TBL1XR1. B. Proteins that have direct interaction with TBL1XR1 in the network of A. It includes 12 nodes and 11 edges. The width of the edge represents the combined score of the interaction from the String database. NCOR1 was finally chosen and labeled with red. Structure of the WT (C) and MUT (D) TBL1XR1 in complex with NCOR1. The Hbonds are shown in the form of a green dashed line. Contact residues within 4Å of TBL1XR1 and NCOR1 are presented in stick form. The electrostatic potentials of WT (E) and MUT (F). The electrostatic potential is display from red (-2.0 kT/e) to blue (+2.0 kT/e). Hbond: hydrogen bond; MUT: mutant; PPI: protein-protein interaction; WT: wild type. Figure S3. Molecular docking between GAPDH and NCOR1 as the negative control. The Hbonds are shown in the form of a green dashed line. Contact residues within 4Å of GADPH and NCOR1 are presented in stick form. Hbond: hydrogen bond.

## Data Availability

The initial sequencing data that backs the discoveries of this research are subject to the limitations set by the Ethics Committee of the First Medical Center of the PLA General Hospital so as to safeguard patient privacy. The data can be obtained from the corresponding author Guang Yang (yangg301@126.com) by researchers who fulfill the requirements for accessing confidential data.

## Abbreviations

ABC: Autism behavior checklist
ACMG: American College of Medical Genetics
ASD: Autism spetrum disorder
bp: Base pairs
CARS: Childhood autism rating scale
cm: Centimeter
CNV: Copy number variation
DD: Developmental delay
DQ: Developmental quotient
EEG: Electroencephalogram
ELE: Electrostatic energy
F: Father
GB: Generalized born energy
GDD: Global developmental delay
GDS: Gesell developmental schedules
GMQE: Global model quality estimation
GRCh38: Genome Reference Consortium Human 38
Hbonds: Hydrogen bonds
HEK293T: Human epithelial kidney 293T
ID: Intellectual disability
Indels: Insertions and deletions
*K*d: Dissociation constant
kg: Kilogram
M: Mother
m: Months
MD: Molecular dynamics
MRD-41: Autosomal dominant intellectual developmental disorder-41
MRI: Magnetic resonance imaging
MUT: Mutant
NA: Not Available
NC: Negative control
NCSVs: Non-canonical splicing variants
N-CoR: Nuclear receptor corepressor
NDDs: Neurodevelopmental disorders
P1/2: Patient 1/2
PDB: Protein data bank
Pre-mRNAs: Precursor messenger RNAs
PPI: Protein–protein interaction
PS: Pierpont syndrome
QMEAN: Qualitative model energy analysis
Rg: Radius of gyration
RMSD: Root-mean-square deviation
RMSF: Root-mean-square fluctuation
RT-PCR: Reverse transcription-polymerase chain reaction
SA: Solvent accessible surface area energy
SASA: Solvent-accessible surface areas
SAXS: Small-angle X-ray scattering
SMRT: Silencing mediator of retinoic acid and thyroid hormone receptor
SNP: Single Nucleotide Polymorphism
TOTAL: Binding free energy
VDW: Van der Waals energy
VUS: Variant of uncertain significance
WES: Whole exome sequencing
WT: Wild-type
y: Years old
Δi*G*: Solvation free energy gain
Δ*G*: Binding free energy

## References

[1] Zhang XM, Chang Q, Zeng L, Gu J, Brown S, Basch RS. TBLR1 regulates the expression of nuclear hormone receptor co-repressors. BMC Cell Biol. 2006;7:31. 16893456 10.1186/1471-2121-7-31 PMC1555579

[2] Oberoi J, Fairall L, Watson PJ, Yang JC, Czimmerer Z, Kampmann T, et al. Structural basis for the assembly of the SMRT/NCoR core transcriptional repression machinery. Nat Struct Mol Biol. 2011;18(2):177–84. 21240272 10.1038/nsmb.1983 PMC3232451

[3] Li J, Wang CY. TBL1-TBLR1 and beta-catenin recruit each other to Wnt target-gene promoter for transcription activation and oncogenesis. Nat Cell Biol. 2008;10(2):160–9. 18193033 10.1038/ncb1684

[4] Pierpont ME, Stewart FJ, Gorlin RJ. Plantar lipomatosis, unusual facial phenotype and developmental delay: a new MCA/MR syndrome. Am J Med Genet. 1998;75(1):18–21. 9450851

[5] Heinen CA, Jongejan A, Watson PJ, Redeker B, Boelen A, Boudzovitch-Surovtseva O, et al. A specific mutation in TBL1XR1 causes Pierpont syndrome. J Med Genet. 2016;53(5):330–7. 26769062 10.1136/jmedgenet-2015-103233 PMC4853543

[6] Arroyo Carrera I, Fernández-Burriel M, Lapunzina P, Tenorio JA, García Navas VD, Márquez Isidro E. TBL1XR1 associated intellectual disability, a new missense variant with dysmorphic features plus autism: expanding the phenotypic spectrum. Clin Genet. 2021;99(6):812–7. 33527360 10.1111/cge.13937

[7] Pons L, Cordier MP, Labalme A, Till M, Louvrier C, Schluth-Bolard C, et al. A new syndrome of intellectual disability with dysmorphism due to TBL1XR1 deletion. Am J Med Genet A. 2015;167a(1):164–8. 25425123 10.1002/ajmg.a.36759

[8] O’Roak BJ, Vives L, Fu W, Egertson JD, Stanaway IB, Phelps IG, et al. Multiplex targeted sequencing identifies recurrently mutated genes in autism spectrum disorders. Science. 2012;338(6114):1619–22. 23160955 10.1126/science.1227764 PMC3528801

[9] O’Roak BJ, Vives L, Girirajan S, Karakoc E, Krumm N, Coe BP, et al. Sporadic autism exomes reveal a highly interconnected protein network of de novo mutations. Nature. 2012;485(7397):246–50. 22495309 10.1038/nature10989 PMC3350576

[10] Quan Y, Zhang Q, Chen M, Wu H, Ou J, Shen Y, et al. Genotype and phenotype correlations for TBL1XR1 in neurodevelopmental disorders. J Mol Neurosci. 2020;70(12):2085–92. 32524419 10.1007/s12031-020-01615-7

[11] Firth HV, Richards SM, Bevan AP, Clayton S, Corpas M, Rajan D, et al. DECIPHER: database of chromosomal imbalance and phenotype in humans using Ensembl resources. Am J Hum Genet. 2009;84(4):524–33. 19344873 10.1016/j.ajhg.2009.03.010 PMC2667985

[12] Nagy A, Molay F, Hargadon S, Brito Pires C, Grant N, De La Rosa AL, et al. The spectrum of neurological presentation in individuals affected by TBL1XR1 gene defects. Orphanet J Rare Dis. 2024;19(1):79. 38378692 10.1186/s13023-024-03083-3 PMC10880200

[13] Baralle M, Baralle FE. The splicing code. Biosystems. 2018;164:39–48. 29122587 10.1016/j.biosystems.2017.11.002

[14] Dufner-Almeida LG, do Carmo RT, Masotti C, Haddad LA. Understanding human DNA variants affecting pre-mRNA splicing in the NGS era. Adv Genet. 2019;103:39–90. 30904096 10.1016/bs.adgen.2018.09.002

[15] Bao S, Moakley DF, Zhang C. The splicing code goes deep. Cell. 2019;176(3):414–6. 30682368 10.1016/j.cell.2019.01.013

[16] Song J, Zhu Y. Children’s neuropsychological tests. Shanghai: Shanghai Scientific and Technological Publishing Company; 1987.

[17] Yang Y. Rating scales for children’s developmental behavior and mental health. Beijing: People’s Medical Publishing House; 2016. p. 369–71.

[18] Liu ZH, Li YR, Lu YL, Chen JK. Clinical research on intelligence seven needle therapy treated infants with brain damage syndrome. Chin J Integr Med. 2016;22(6):451–6. 26085198 10.1007/s11655-015-1977-9

[19] Choi Y, Sims GE, Murphy S, Miller JR, Chan AP. Predicting the functional effect of amino acid substitutions and indels. PLoS ONE. 2012;7(10):e46688. 23056405 10.1371/journal.pone.0046688 PMC3466303

[20] Laskowski RA, Jabłońska J, Pravda L, Vařeková RS, Thornton JM. PDBsum: structural summaries of PDB entries. Protein Sci. 2018;27(1):129–34. 28875543 10.1002/pro.3289 PMC5734310

[21] Jumper J, Evans R, Pritzel A, Green T, Figurnov M, Ronneberger O, et al. Highly accurate protein structure prediction with AlphaFold. Nature. 2021;596(7873):583–9. 34265844 10.1038/s41586-021-03819-2 PMC8371605

[22] Waterhouse A, Bertoni M, Bienert S, Studer G, Tauriello G, Gumienny R, et al. SWISS-MODEL: homology modelling of protein structures and complexes. Nucleic Acids Res. 2018;46(W1):W296-w303. 29788355 10.1093/nar/gky427 PMC6030848

[23] Cardoso JMS, Fonseca L, Egas C, Abrantes I. Cysteine proteases secreted by the pinewood nematode, *Bursaphelenchus xylophilus*: in silico analysis. Comput Biol Chem. 2018;77:291–6. 30399505 10.1016/j.compbiolchem.2018.10.011

[24] Studer G, Rempfer C, Waterhouse AM, Gumienny R, Haas J, Schwede T. QMEANDisCo-distance constraints applied on model quality estimation. Bioinformatics. 2020;36(6):1765–71. 31697312 10.1093/bioinformatics/btz828 PMC7075525

[25] Carugo O, Djinovic-Carugo K. Half a century of Ramachandran plots. Acta Crystallogr D Biol Crystallogr. 2013;69(Pt 8):1333–41. 23897457 10.1107/S090744491301158X

[26] DeLano WL. Pymol: an open-source molecular graphics tool. CCP4 Newsl protein crystallogr. 2002;40(1):82–92.

[27] LLC. The PyMOL Molecular Graphics System. Version 26 Schrödinger.

[28] Rakhshani H, Dehghanian E, Rahati A. Enhanced GROMACS: toward a better numerical simulation framework. J Mol Model. 2019;25(12):355. 31768713 10.1007/s00894-019-4232-z

[29] Wang J, Wang W, Kollman PA, Case DA. Automatic atom type and bond type perception in molecular mechanical calculations. J Mol Graph Model. 2006;25(2):247–60. 16458552 10.1016/j.jmgm.2005.12.005

[30] Petersen HG. Accuracy and efficiency of the particle mesh Ewald method. J Chem Phys. 1995;103(9):3668–79.

[31] Stelzer G, Plaschkes I, Oz-Levi D, Alkelai A, Olender T, Zimmerman S, et al. VarElect: the phenotype-based variation prioritizer of the GeneCards Suite. BMC Genomics. 2016;17:444. 27357693 10.1186/s12864-016-2722-2 PMC4928145

[32] Shannon P, Markiel A, Ozier O, Baliga NS, Wang JT, Ramage D, et al. Cytoscape: a software environment for integrated models of biomolecular interaction networks. Genome Res. 2003;13(11):2498–504. 14597658 10.1101/gr.1239303 PMC403769

[33] Abramson J, Adler J, Dunger J, Evans R, Green T, Pritzel A, et al. Accurate structure prediction of biomolecular interactions with AlphaFold 3. Nature. 2024;630(8016):493–500. 38718835 10.1038/s41586-024-07487-w PMC11168924

[34] Kapusta K, McGowan A, Banerjee S, Wang J, Kolodziejczyk W, Leszczynski J. Benchmark investigation of SARS-CoV-2 mutants’ immune escape with 2B04 murine antibody: a step towards unraveling a larger picture. Curr Issues Mol Biol. 2024;46(11):12550–73. 39590339 10.3390/cimb46110745 PMC11592782

[35] Weng G, Wang E, Wang Z, Liu H, Zhu F, Li D, et al. HawkDock: a web server to predict and analyze the protein-protein complex based on computational docking and MM/GBSA. Nucleic Acids Res. 2019;47(W1):W322-w330. 31106357 10.1093/nar/gkz397 PMC6602443

[36] Krissinel E. Crystal contacts as nature’s docking solutions. J Comput Chem. 2010;31(1):133–43. 19421996 10.1002/jcc.21303

[37] Krissinel E, Henrick K. Inference of macromolecular assemblies from crystalline state. J Mol Biol. 2007;372(3):774–97. 17681537 10.1016/j.jmb.2007.05.022

[38] Xue LC, Rodrigues JP, Kastritis PL, Bonvin AM, Vangone A. PRODIGY: a web server for predicting the binding affinity of protein–protein complexes. Bioinformatics. 2016;32(23):3676–8. 27503228 10.1093/bioinformatics/btw514

[39] Vangone A, Bonvin AMJJ. Contacts-based prediction of binding affinity in protein–protein complexes. Elife. 2015;4:e07454. 26193119 10.7554/eLife.07454 PMC4523921

[40] Li Q, Wang K. InterVar: clinical interpretation of genetic variants by the 2015 ACMG-AMP guidelines. Am J Hum Genet. 2017;100(2):267–80. 28132688 10.1016/j.ajhg.2017.01.004 PMC5294755

[41] Richards S, Aziz N, Bale S, Bick D, Das S, Gastier-Foster J, et al. Standards and guidelines for the interpretation of sequence variants: a joint consensus recommendation of the American College of Medical Genetics and Genomics and the Association for Molecular Pathology. Genet Med. 2015;17(5):405–24. 25741868 10.1038/gim.2015.30 PMC4544753

[42] Yoon HG, Chan DW, Huang ZQ, Li J, Fondell JD, Qin J, et al. Purification and functional characterization of the human N-CoR complex: the roles of HDAC3, TBL1 and TBLR1. EMBO J. 2003;22(6):1336–46. 12628926 10.1093/emboj/cdg120 PMC151047

[43] Chen VB, Arendall WB 3rd, Headd JJ, Keedy DA, Immormino RM, Kapral GJ, et al. MolProbity: all-atom structure validation for macromolecular crystallography. Acta Crystallogr D Biol Crystallogr. 2010;66(Pt 1):12–21. 20057044 10.1107/S0907444909042073 PMC2803126

[44] Wu XH, Wang Y, Zhuo Z, Jiang F, Wu YD. Identifying the hotspots on the top faces of WD40-repeat proteins from their primary sequences by β-bulges and DHSW tetrads. PLoS ONE. 2012;7(8):e43005. 22916195 10.1371/journal.pone.0043005 PMC3419727

[45] Wei L, Yang Y, Jiang T, Zhang C, Chen C, Huang M, et al. Different mutations in *TBL1XR1* lead to diverse phenotypes of neurodevelopmental disorder: two case reports. BMC Med Genomics. 2025;18(1):96. 40426223 10.1186/s12920-025-02169-6 PMC12117794

[46] Tabet AC, Leroy C, Dupont C, Serrano E, Hernandez K, Gallard J, et al. De novo deletion of TBL1XR1 in a child with non-specific developmental delay supports its implication in intellectual disability. Am J Med Genet A. 2014;164a(9):2335–7. 24891185 10.1002/ajmg.a.36619

[47] Ou Z, Liu G, Liu W, Deng Y, Zheng L, Zhang S, et al. Bioinformatics analysis of CYP1B1 mutation hotspots in Chinese primary congenital glaucoma patients. Biosci Rep. 2018; 10.1042/bsr20180056 29903728 10.1042/BSR20180056 PMC6435531

[48] Mastrototaro G, Zaghi M, Massimino L, Moneta M, Mohammadi N, Banfi F, et al. TBL1XR1 ensures balanced neural development through NCOR complex-mediated regulation of the MAPK pathway. Front Cell Dev Biol. 2021;9:641410. 33708771 10.3389/fcell.2021.641410 PMC7940385

[49] Hu Y, Falize K, van Trotsenburg ASP, Hennekam R, Fliers E, Bruinstroop E, et al. The role of transducin β-like 1 X-linked receptor 1 (TBL1XR1) in thyroid hormone metabolism and action in mice. Eur Thyroid J. 2023;12(5):e230077. 37458724 10.1530/ETJ-23-0077 PMC10448563

[50] Hu Y, Lauffer P, Stewart M, Codner G, Mayerl S, Heuer H, et al. An animal model for Pierpont syndrome: a mouse bearing the Tbl1xr1Y446C/Y446C mutation. Hum Mol Genet. 2022;31(17):2951–63. 35416977 10.1093/hmg/ddac086 PMC9433735

[51] Laskowski RA, Tyagi N, Johnson D, Joss S, Kinning E, McWilliam C, et al. Integrating population variation and protein structural analysis to improve clinical interpretation of missense variation: application to the WD40 domain. Hum Mol Genet. 2016;25(5):927–35. 26740553 10.1093/hmg/ddv625 PMC4754046

[52] Slavotinek A, Pua H, Hodoglugil U, Abadie J, Shieh J, Van Ziffle J, et al. Pierpont syndrome associated with the p.Tyr446Cys missense mutation in TBL1XR1. Eur J Med Genet. 2017;60(10):504–8. 28687524 10.1016/j.ejmg.2017.07.003

