# Supplementary material for: Two cases of TBL1XR1 heterozygous variants in children: a new splicing site variant identification and functional analysis through molecular docking and molecular dynamics simulation
Source: Hum Genomics. 2025 Dec 24;20:8. doi: 10.1186/s40246-025-00877-9 (PMC12781238; doi:10.1186/s40246-025-00877-9)
Supplement: Supplementary file 5 — Supplementary material 5: Figure S1. Two splice patterns predicted by RDDC tool. Pattern 1 with 6 bp insertion and pattern 2 with exon 10 (61 bp) skipping. bp: base pairs. Figure S2. Molecular docking between TBL1XR1 and NCOR1. A. Interactive PPI network obtained from Genecards and String databases. It includes 111 nodes and 371 edges. Each node represents PS-related proteins, each edge represents the associated interaction. Pale green indicates proteins that have indirect interaction with TBL1XR1; light yellow indicates proteins that have direct interaction with TBL1XR1; orange indicates TBL1XR1. B. Proteins that have direct interaction with TBL1XR1 in the network of A. It includes 12 nodes and 11 edges. The width of the edge represents the combined score of the interaction from the String database. NCOR1 was finally chosen and labeled with red. Structure of the WT (C) and MUT (D) TBL1XR1 in complex with NCOR1. The Hbonds are shown in the form of a green dashed line. Contact residues within 4Å of TBL1XR1 and NCOR1 are presented in stick form. The electrostatic potentials of WT (E) and MUT (F). The electrostatic potential is display from red (-2.0 kT/e) to blue (+2.0 kT/e). Hbond: hydrogen bond; MUT: mutant; PPI: protein-protein interaction; WT: wild type. Figure S3. Molecular docking between GAPDH and NCOR1 as the negative control. The Hbonds are shown in the form of a green dashed line. Contact residues within 4Å of GADPH and NCOR1 are presented in stick form. Hbond: hydrogen bond. [file 40246_2025_877_MOESM5_ESM.docx]

Supplemental Data


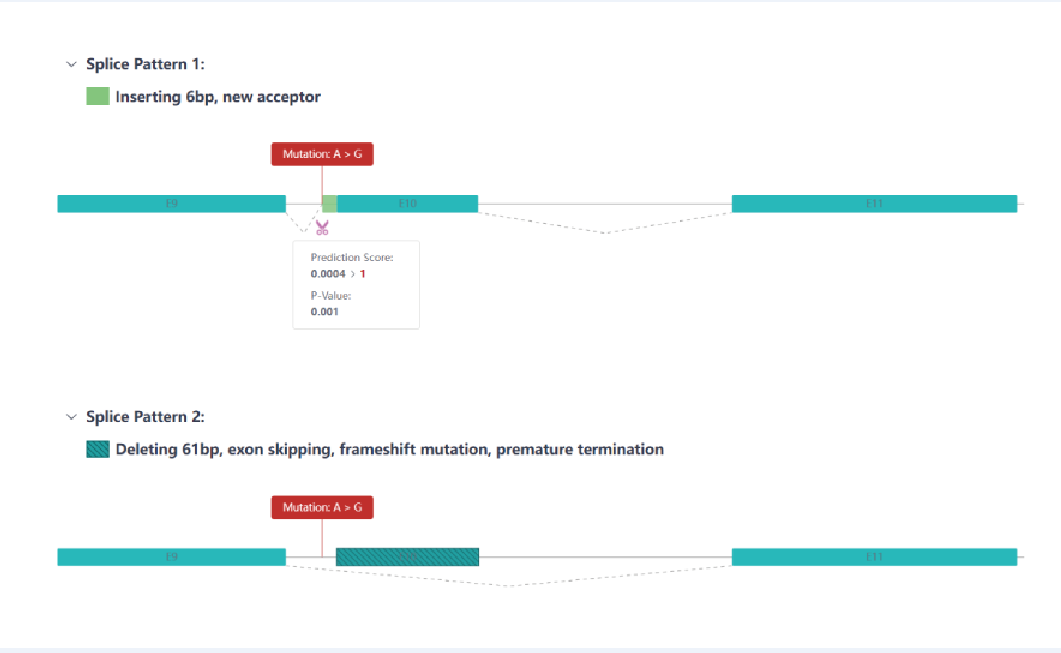


**Figure S1.** Two splice patterns predicted by RDDC tool.

Pattern 1 with 6 bp insertion and pattern 2 with exon 10 (61 bp) skipping. bp: base pairs.


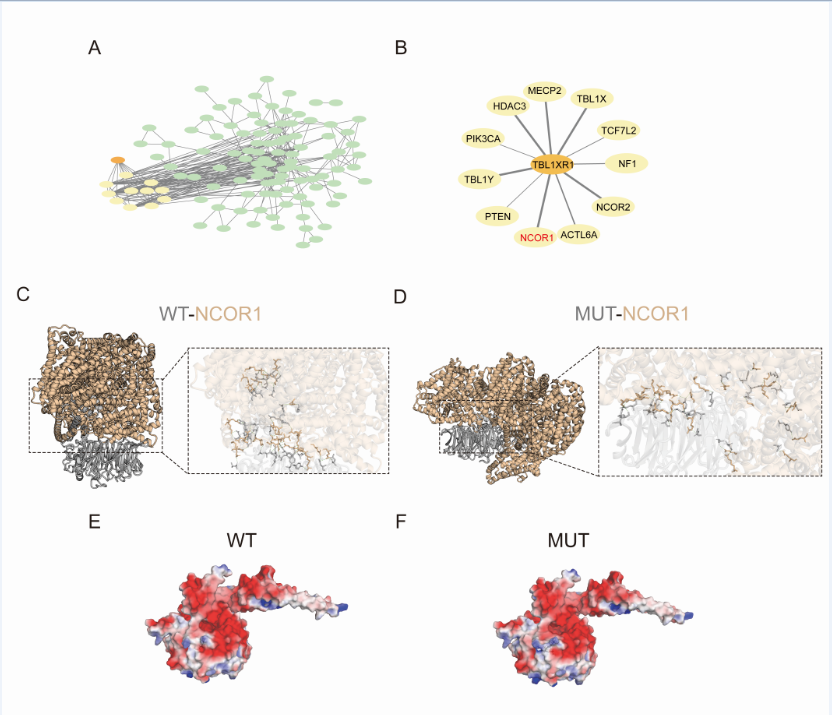


**Figure S2**. **Molecular docking between TBL1XR1 and NCOR1.**

A. Interactive PPI network obtained from Genecards and String databases. It includes 111 nodes and 371 edges. Each node represents PS-related proteins, each edge represents the associated interaction. Pale green indicates proteins that have indirect interaction with TBL1XR1; light yellow indicates proteins that have direct interaction with TBL1XR1; orange indicates TBL1XR1.

B. Proteins that have direct interaction with TBL1XR1 in the network of A. It includes 12 nodes and 11 edges. The width of the edge represents the combined score of the interaction from the String database. NCOR1 was finally chosen and labeled with red.

Structure of the WT (C) and MUT (D) TBL1XR1 in complex with NCOR1. The Hbonds are shown in the form of a green dashed line. Contact residues within 4Å of TBL1XR1 and NCOR1 are presented in stick form.

The electrostatic potentials of WT (E) and MUT (F). The electrostatic potential is display from red (-2.0 kT/e) to blue (+2.0 kT/e).

Hbond: hydrogen bond; MUT: mutant; PPI: protein-protein interaction; WT: wild type.

**
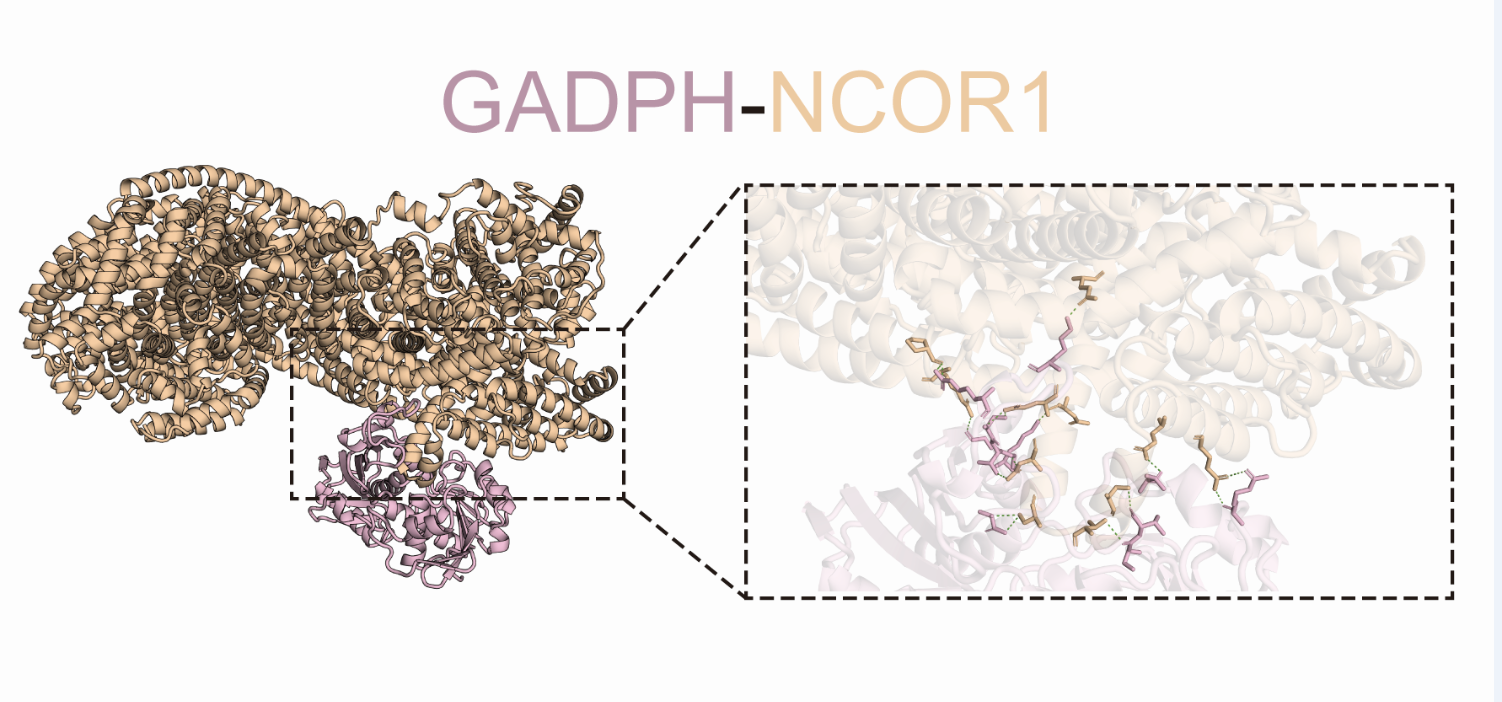
**

**Figure S3. Molecular docking between GAPDH and NCOR1 as the negative control.** The Hbonds are shown in the form of a green dashed line. Contact residues within 4Å of GADPH and NCOR1 are presented in stick form.

Hbond: hydrogen bond.
